# Interactions of sclerostin with FGF23, soluble klotho and vitamin D in renal transplantation

**DOI:** 10.1371/journal.pone.0178637

**Published:** 2017-05-30

**Authors:** Lida Tartaglione, Marzia Pasquali, Silverio Rotondi, Maria Luisa Muci, Cristiana Leonangeli, Alessio Farcomeni, Valeria Fassino, Sandro Mazzaferro

**Affiliations:** 1 Department of Cardiovascular, Respiratory, Nephrologic, Anesthesiologic and Geriatric Sciences, Sapienza University of Rome, Italy; 2 Department of Nephrology and Dialysis, University Hospital Company, Policlinico Umberto I, Rome, Italy; 3 Department of Public Health and Infectious Diseases, Sapienza University of Rome, Italy; 4 Department of Internal Medicine and medical Specialties, University Hospital Company, Policlinico Umberto I, Rome, Italy; Hospital Universitario de la Princesa, SPAIN

## Abstract

Relationships of Sclerostin, a bone anti-anabolic protein, with biomarkers of mineral bone disorders in chronic kidney disease are still unsettled, in particular in kidney transplant (KTR). In 80 KTR patients (31F/49M, 54.7±10.3 years) we studied the relationships of serum Sclerostin with eGFR, Calcium, Phosphate, Alkaline Phosphatase (AP), intact Parathyroid hormone (iPTH), soluble alpha-Klotho (sKlotho), intact Fibroblast Growth Factor 23 (iFGF23), 25-hydroxyvitamin D(25D) and 1,25-dihydroxyvitamin D (1,25D). Thirty healthy subjects (35.0±12.4 years, eGFR 109.1±14.1 ml /min/1,73m^2^) served as control for Sclerostin, iFGF23 and sKlotho. With a median eGFR of 46.3 mL/min/1.73m^2^ (IQR, 36.2–58.3) our KTR had median Sclerostin levels of 23.7 pmol/L (IQR: 20.8–32.8), not different from controls (26.6 pmol/L, IQR: 22.0–32.2; p = n.s). Sclerostin correlated negatively with AP (r = -.251; p = 0.023) and positively with iFGF23 (r = .227; p = 0.017) and 25D (r = .214; p = 0.025). Age-adjusted multiple regression analysis identified AP and 1,25D as negative and 25D and sKlotho as positive best predictors of Sclerostin. No correlation was evident with eGFR. The negative correlation with AP confirms the direct anti-anabolic role of Sclerostin. The associations either negative or positive with iFGF23, sKlotho, and vitamin D metabolites suggest also a modulatory role in mineral homeostasis. In particular, the associations with iFGF23 (positive) and 1,25D (negative) underline the relevant inhibitory action of Sclerostin on vitamin D activation. In conclusion, Sclerostin levels in KTR are normal and influenced more by bone turnover than by eGFR. Its involvement with other hormones of mineral homeostasis (FGF23/Klotho and Vitamin D) is part of the sophisticated cross-talk between bone and the kidney.

## Introduction

Produced by osteocytes, Sclerostin is a powerful modulator of bone modeling and remodeling and do this by inhibiting the canonical Wnt (Wingless-type mouse mammary tumor virus integration site) pathway. Wnt receptor inhibition by Sclerostin reduces osteoblastogenesis and promotes osteoblast and osteocyte apoptosis thus exerting a powerful anti-anabolic effect. Moreover, since Sclerostin also induces RANKL synthesis and thus stimulates osteoclastogenesis, its inhibition both increases osteoblast and reduces osteoclast activity, with an overall increment of bone formation and mass [1,2] and eventual osteosclerosis [3]. Indeed, Sclerostin gene (SOST) knock-out mice and SOST inactivating mutations in humans [4,5], are both characterized by increased bone formation and sclerosis of the skeleton [6]. Recently, also indirect effects of Sclerostin on mineral metabolism are envisaged, since Sclerostin can increase the activity of Fibroblast growth Factor 23 (FGF23), a bone protein with powerful phosphate and vitamin D regulating properties [7]. Thus, Sclerostin promises to become an important element of the bone-kidney axis represented up to now by FGF23 and its kidney-produced co-receptor Klotho [8]. For these reasons, recent clinical studies aim to evaluate Sclerostin as a new biomarker of bone disease specifically in chronic kidney disease (CKD). In non-dialysis CKD, observational studies show that serum levels of Sclerostin increase along with the stages of renal failure [9,10] and correlate positively with serum FGF23 and phosphate [9,11]. Renal function reduction does not seem to affect its serum levels since also tubular excretion increases [12]. In dialysis patients (CKD-5D), Sclerostin levels are increased and similarly correlated positively with FGF23 and phosphate. Further in dialysis, negative relationships are described with parathyroid hormone (PTH), alkaline phosphatase (AP) and bone turnover [13–16]. In renal transplant patients (KTR), factors like immunosuppressive therapy, pre-existing bone lesion and/or the presence of variable degrees of reduced renal function may affect mineral and bone disorders (MBD). Thus, in these patients the diagnostic significance of Sclerostin serum levels warrants specific evaluation. The few available studies describe a return to the normal range early after transplantation [17] and, later on, the presence of a negative correlation with eGFR, PTH and 1,25-dihydroxyvitaminD [18]. However, in post-menopausal KTR females, no correlation has been found with biomarkers of bone resorption or formation [19].

Notably, the pathophysiologic links of Sclerostin with MBD in CKD have been recently claimed to explain the association with vascular calcification, cardiovascular disease and mortality [14,18,20], thus further increasing the clinical interest for this new biomarker.

Since clinical data in KTR are still limited, we considered useful to evaluate the relationship of Sclerostin serum levels with renal function and the hormonal systems that are potentially involved with its activity, namely FGF23/Klotho and vitamin D.

## Materials and methods

### Patients

We enrolled in a cross-sectional study, 80 renal transplant patients from our outpatient unit. Inclusion criteria were: age 18–80 years, estimated GFR (eGFR) > 15 mL/min/1.73m^2^, time since transplant ≥1 year, no evidence of acute underlying illness.

Exclusion criteria were: comorbid conditions such as cancer, liver disease or any severe systemic disease that might affect data interpretation. If any, treatment with native or active vitamin D was withhold for 3 months prior to the study. Further none of the patients required therapy with calcimimetic or phosphate binders.

Fasting blood samples were obtained for the measurement of creatinine (Cr), Calcium (Ca), Phosphate (P), total alkaline phosphatase (AP), serum collagen type 1 cross-linked C-telopeptide (CTX), intact parathyroid hormone (iPTH), 25-hydroxyvitamin D (25D), 1,25-dihydroxyvitamin D (1,25D), intact FGF23 (iFGF23), soluble alpha-Klotho (sKlotho) and Sclerostin. We also collected fasting spot urine samples from all participants at the time of blood sampling to measure creatinine, phosphate, and calcium. In each patient, we recorded clinical parameters and prescribed therapies.

Thirty healthy subjects (mean age 35.0 ± 12.4 years, eGFR 109.1±14.1 ml /min/1,73m^2^) served as control to obtain local reference values for non-routinely assayed Sclerostin, sKlotho and iFGF23.

The present study is a subanalysis of the "Studio 25051954—EudraCT 2010-021041-42", approved by the Ethical Committee of the Azienda Policlinico Umberto I, and was performed in accordance with the declaration of Helsinky. Written informed consent was obtained from all patients. None of the transplant donors were from a vulnerable population and all donors or next of kin provided written informed consent that was freely given.

### Assays

Routine blood analytes (creatinine, Ca, P and AP) were immediately assayed. Samples for non-routine assay of iPTH, Sclerostin, iFGF23, sKlotho, 25D and 1,25D were immediately frozen and stored at -30°C until measurement.

Standard colorimetric or enzymatic techniques were kinetic alkaline picrate (creatinine), cresolphthalein-complexone (Ca), ammonium molybdate (P) and p-nitrophenylphosphate (AP). The reference range of AP values is within 80 and 270 IU/ml.

iPTH was an immunoradiometric technique (DiaSorin, Stillwater, MN, USA) based on a double antibody against the intact molecule; our normal values are within 10–55 pg/mL, with intra- and inter-assay variations of 6.5% and 9.8%, respectively.

Serum 25Dwas assayed with a commercial kit (DiaSorin, Stillwater, MN, USA) that included sample purification with acetonitrile followed by a ^125^I-based radioimmunoassay. Intra- and inter-assay coefficients of variation are10.8% and 9.4%, respectively.

Levels of 1,25D were measured with a radioimmunoassay according to the manufacturer’s protocol (IDS Ltd, Bolton, UK) including a monoclonal immune-extraction, followed by quantitation with a standard ^125^I-based radioimmunoassay. Intra- and inter-assay coefficients of variation are <12% and <14%, respectively. The normal range observed in our laboratory was between 19.5 and 67.0 pg/mL.

iFGF23 was assayed with a commercially available kit (Kainos Lab. Inc. Tokyo, Japan) that utilizes a two-site ELISA for the full-length molecule. Two specific murine monoclonal antibodies recognized the biologically active FGF23, with a lower limit of detection of 3 pg/ml, and inter- and intra-assay coefficients of variation of <5%.

Serum levels of soluble alpha-Klotho were assayed with an enzyme-linked immunosorbent assay (ELISA) method that utilizes a monoclonal antibody with strong affinity for Klotho protein, recognizing with high selectivity the tertiary protein structure of its extracellular domain (Immuno-Biological Laboratories Co., Ltd.). Within- and between-run variation of the alpha-Klotho IBL was <5 and<8%, respectively.

Serum Sclerostin was a commercially available kit (Biomedica gruppe, Vienna, Austria) that utilizes a sandwich ELISA for the quantitative determination of human Sclerostin as previously reported [21]. Inter- and intra-assay variation coefficients were<10% and <7% respectively. The detection limit of the Sclerostin ELISA was 3.2 pmol/L.

Serum Collagen type 1 cross-linked C-telopeptide (CTX) was a commercially available kit (Immunodiagnostic systems, Boldon, UK) employing an ELISA test with monoclonal murine antibody specific for degradation products of C-terminal telopeptides of Type I collagen. Inter- and Intra-assay coefficients of variation were <10.9% and <3.0%.

Glomerular filtration rate (GFR) was estimated according to the Chronic Kidney Disease Epidemiology Collaboration (CKD-EPI) equation (eGFR = 141 x min (Scr/κ, 1)^α^ x max (Scr/κ,1)^-1.209^ x 0.993^Age^ x 1.018 [if female] x 1.159 [if black]) [22].

### Statistical analysis

Data are expressed as mean±SD for Gaussian variables or median and IQR when normality was not tenable. We used Kolmogorov-Smirnov test to evaluate normality of continuous measurements and Pearson correlation to assess linear covariation of Gaussian measurements. Log-measurements were confirmed to be normally distributed and were used as outcomes in multivariate regression models. The final multivariate model was obtained by minimizing the Akaike information criterion via a forward stepwise regression. All tests are two tailed and (adjusted)-values <0.05 were considered as statistically significant. Analyses were performed using the open source software package R version 3.1.2.

## Results

Main clinical and biochemical data of our population of patients, which included 31 women and 49 men aged 54.7±10.3 (M±SD) years, are shown in Table 1. Median eGFR was 46.3 mL/min/1.73m^2^ (IQR, 36.2–58.3) with CKD stages ranging within 2 and 4, while median time since transplantation was 77.6 (IQR, 37.6–119.5) months (range 12–268 months).

**Table 1:** Clinical and biochemical characteristics of the population under study.

Age (y)	55.7 (47.9–62.1)
Gender (male/female)	49 (61.2%)/31(38.8%)
B.M.I.	24.1(22.2–27.3)
Time from transplantation (months)	77.6 (37.6–119.5)
eGFR (ml/min/1.73m^2^)	46.3 (36.2–58.3)
Calcium (mg/dl)	10.1 (9.7–10.5)
Phosphate (mg/dl)	2.9 (2.5–3.5)
iPTH (pg/ml)	43.1(25.4–70.6)
25-hydroxyvitaminD (ng/ml)	25.1 (16.9–35.1)
1,25-dihydroxyvitaminD (pg/ml)	41.9 (30.1–53.1)
Alkaline phosphatase (U/L) [NV 80–270]	187.0 (139.1–221.0)
CTX, ng/ml	0.553 (0.310–0.816)
Ca_u_/Cr_u_, mg/mg	0.050 (0.027–0.098)
FE PO_4_, %	28.9 (20.0–40.7)
**Therapies**	
Steroids	59 (73.75%)
Calcineurin inhibitors	73 (91.25%)
Proliferationsignalinhibitors	5 (6.25%)
Anti-metabolite	65 (81.25%)

Note: Data are expressed as median (interquartile range). Categorical data are presented as numbers (percentages).

Abbreviations: BMI, body mass index; eGFR, estimated glomerular filtration rate; iPTH, intact parathyroid hormone; CTX,collagen type 1 cross-linked C-telopeptide; Ca_u_/Cr_u_, calcium/creatinine ratio; FE PO_4_, fractional excretion of phosphate.

Median serum levels of calcium (10.1 mg/dl, IQR: 9.7–10.5), phosphate (2.9 mg/dl, IQR: 2.5–3.5), alkaline phosphatase (187.0 U/L; IQR: 139.1–221.0), CTX 0.553 ng/ml (IQR: 0.310–0.816) and iPTH (43.1 pg/ml, IQR: 25.4–70.6) were all within the normal ranges. Serum level of 1,25D (41.9 pg/ml, IQR: 30.1–53.1) averaged normal values, in front of mildly reduced 25D levels (25.1 ng/ml, IQR: 16.9–35.1). Median calcium/creatinine ratio (Ca_u_/Cr_u_) and fractional excretion of phosphate (FE PO_4_) were, respectively, 0,050 (IQR: 0,027–0,098 and 28.9% (IQR: 20.0–40.7).

**Fig 1:**
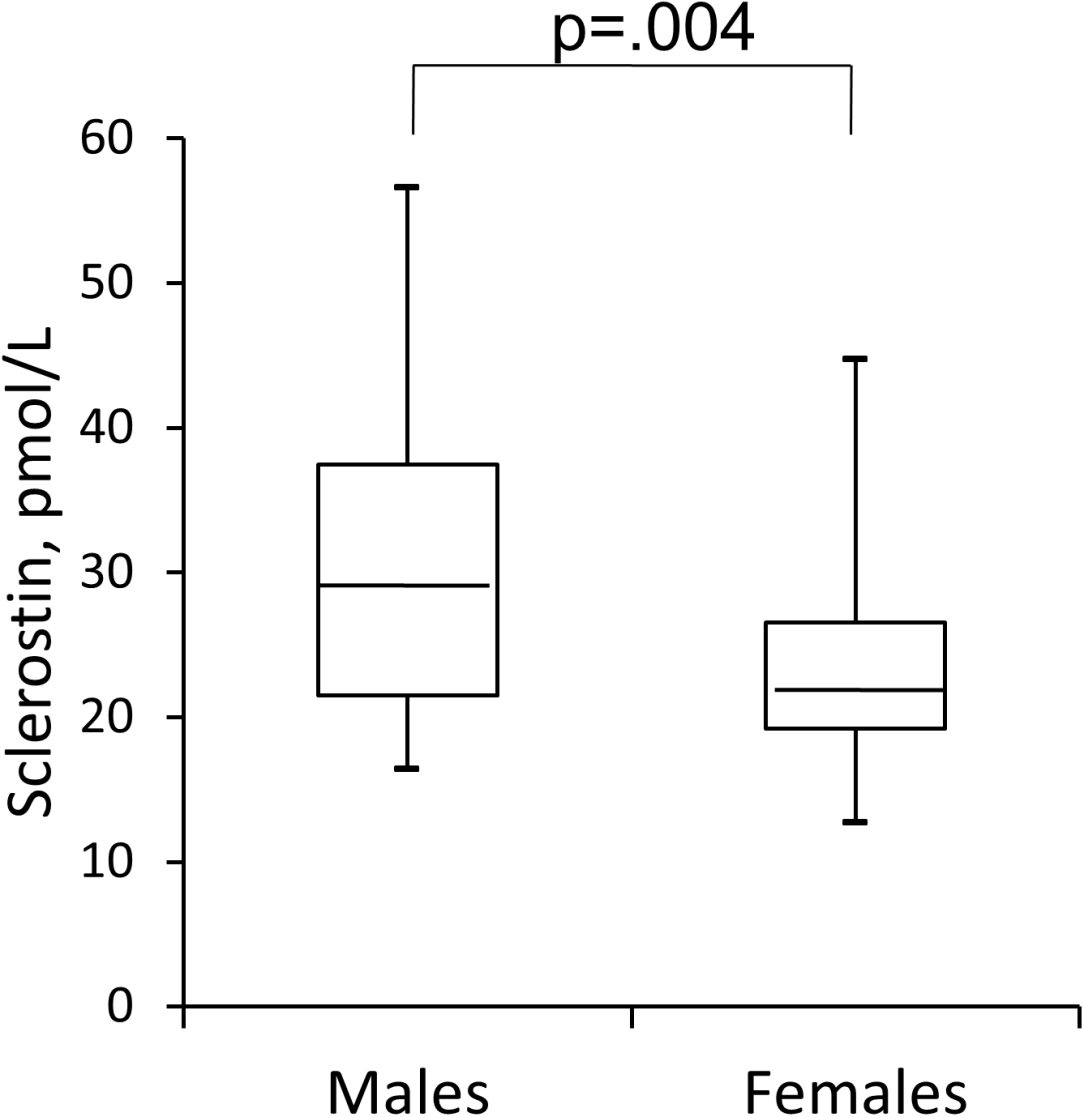
Gender differences in Sclerostin serum levels in renal transplantation. Indicated are medians, first and third quartiles, minimal and maximal values. Males versus Females p < .05.

In this population, as shown in Table 2, circulating levels of Sclerostin (23.7 pmol/L, IQR: 20.8–32.8) were not significantly different from the control group (26.6 pmol/L, IQR: 22.0–32.2; p = n.s.). Further, we analyzed Sclerostin levels according to CKD stages but did not find differences (CKD stage 2 = 24.9±11.4; CKD stage 3 = 28.8±10.2; CKD stage 4 = 25.7±9.3; p = .351). No differences were observed between diabetic and non-diabetic patients (respectively 25.9±10.0 vs 28.2 ± 11.0; p = .596). There was however a gender difference, with higher values in males (28.7, IQR:16.4–56.6 vs 21.7, IQR:12.7–44.7 pmol/l; p = 0.004) (Fig 1).

**Table 2:** Serum levels of Sclerostin, FGF23 and Klotho in our control group and renal transplant population.

	KTR	Control Group	P values
Sclerostin (pmol/L)	23.7 (20.8–32.8)	26.6 (22.0–32.2)	0.804
iFGF23 (pg/ml)	40.5 (25.0–59.0)	33.6 (28.1–44.1)	0.003
sKlotho (pg/ml)	449.3 (387.7–533.9)	794.9 (619.4–900.5)	0.001

Note: Data are expressed as median (interquartile range).

Abbreviations: KTR, renal transplant patients; iFGF23, intact Fibroblast Growth Factor 23; sKlotho, soluble alpha Klotho.

Compared to the control group, serum levels of iFGF23 and sKlotho were, respectively, significantly increased (40.5, IQR: 25.0–59.0 vs 33,6, IQR: 28.1–44.2pg/ml; p = 0.003), and significantly decreased (449.3, IQR: 387.7–533.9 vs 794.9, IQR: 619.4–900.5 pg/ml; p = .001) (Table 2).

**Fig 2:**
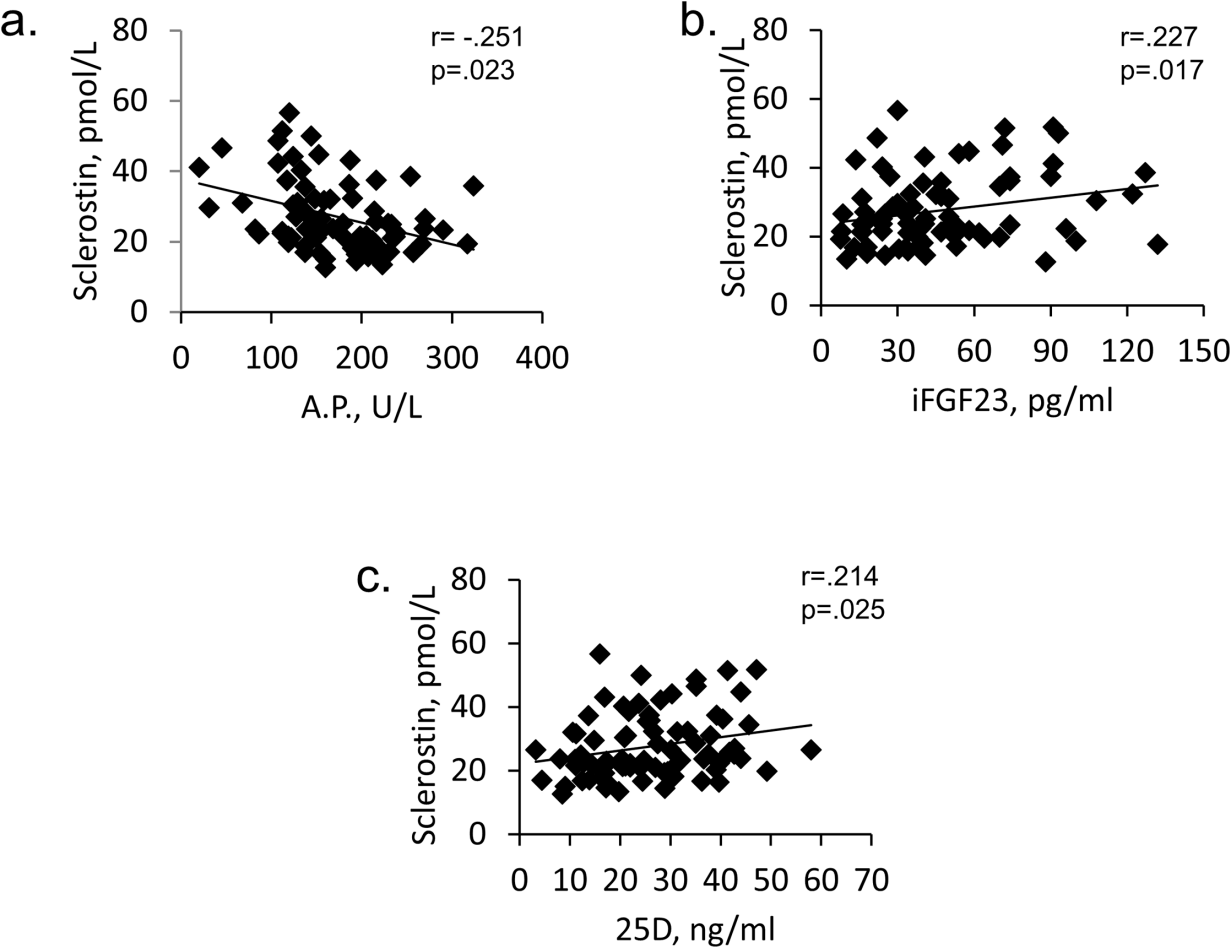
Correlation tests of Sclerostin. Correlation tests with **(**a) AP, Alkaline Phosphatase; (b) iFGF23, intact Fibroblast Growth Factor 23; (c) 25D, 25-hydroxyvitaminD.

Correlation tests showed that Sclerostin correlated negatively with AP (r = -.251; p = 0.023) and positively with iFGF23 (r = .227; p = 0.017) and 25D (r = .214; p = 0.025) (Fig 2A, 2B and 2C), while no correlation was evident with age and eGFR. However, the age-adjusted multi-regression analysis identified AP and 1,25D as negative and 25-D and sKlotho as positive best predictors of Sclerostin (Table 3).

**Table 3:** Age adjusted multivariate analysis with Sclerostin as predicted variable.

VAR	COEF	C.I.	P
Alkaline Phosphatase	-0.036	-0.069, -0.003	0.0337
25-hydroxyvitaminD	0.218	0.0118, 0.424	0.0386
1,25-dihydroxyvitaminD	-0.182	-0.331, -0.034	0.0167
sKlotho	0.017	0.001, 0.032	0.0337

VAR: variable; COEF: coefficient of linear regression; CI: confidence interval; sKlotho, soluble alpha Klotho.

## Discussion

In our KTR population serum Sclerostin levels were not different from the control group and averaged values similar to what has been already published in healthy/general populations [21,23] or in other cohorts of long-term transplant patients [18,19]. Also, the gender difference in our patients (higher values in males) matches the available data in the general population [23] and in renal transplant patients [18] and can be explained by the larger total-body skeletal mass of men [24] and/or by the inhibitory effect of oestrogens on Sclerostin synthesis [24,25].

At variance with previous data in CKD [10] and long term KTR [17, 18], we found no correlation of Sclerostin with eGFR. Nonetheless, our results agree with the hypothesis of increased renal excretion of Sclerostin along with GRF reduction, as described in conservative CKD [10, 12]. Further, the interaction between Sclerostin and eGFR could be masked by differences in bone turnover. In fact, a negative relationship with bone turnover markers has been described both in the general population [21] and in CKD-5D [14,15]. Indeed, also in our KTR serum Sclerostin correlated negatively with AP, an association in agreement with the undisputed inhibitory role on osteoblasts activity. This anti-anabolic role is confirmed by the inverse association with bone specific alkaline phosphatase (bALP) and other markers of bone turnover in non-renal elderly women with presumably limited reduction in GFR and in patients with no residual renal function like hemodialysis patients [21,14,15]. Further, serum Sclerostin has been shown to correlate negatively with osteoblast number and histologic parameters of bone turnover in CKD-5D [13]. To our knowledge, only one paper shows no correlation of Sclerostin with bone resorption and bone formation biomarkers, but in a low number (only 19 cases) of post-menopausal KTR women. Apparently then, the absence of correlation between Sclerostin and eGFR in our study could be explained by possible clinical differences in the enrolled populations. Also, Sclerostin is suggested as a potentially useful biomarker of bone turnover, with no difference between renal and non-renal patients.

Further associations of Sclerostin in our study included the positive linear relationships with iFGF23 and 25D, and the age-adjusted multiple regression analysis indicating AP and 1,25D as negative, and 25D and sKlotho as positive best predictors of Sclerostin.

A positive correlation of Sclerostin with FGF23 has been already described in conservative renal insufficiency [10,11,16] and is confirmatory of the experimental evidence suggesting that Sclerostin increases FGF23 by inhibiting PHEX (a protein encoded by the Phosphate regulating gene with Homologies to Endopeptidases on the X chromosome) [7]. In fact, PHEX protein stimulates FGF23 degradation [26] thus reducing its activity. By interacting with its co-receptor Klotho, FGF23 increases tubular phosphate excretion and inhibits 1,25D synthesis [27,28] with eventual well described clinical effects [8,29]. As a confirmation, Sclerostin knock-out mice have decreased FGF23 with elevated inorganic phosphate concentrations and increased 1,25D levels [26]. Clinical studies, both in pre-dialysis CKD and in dialysis patients, confirm the positive correlation between Sclerostin and FGF23 [18], and a direct correlation has been demonstrated also as bone expression, in a study involving pediatric patients with different types of solid organ allografts (22 patients in total, only 8 KTR) [30]. In adult KTR data are scanty and we are among the few to contemporarily assay serum levels of FGF23 and of Sclerostin. Our results are confirmatory of the physiologic regulatory role of Sclerostin on FGF23 synthesis.

The negative correlation between Sclerostin and 1,25D observed in our study has been already reported in long-term KTR [18] and can be explained with the down regulation of renal 1-alpha-hydroxilase by Sclerostin through direct or indirect (PHEX inhibition and FGF23 stimulation) effects [7]. On the other hand, the inconsistent association of Sclerostin with the two vitamin D metabolites (positive with 25D and negative with 1,25D) is new and needs special consideration. In fact, available clinical observations in healthy and renal patients mostly describe no relationship with 25D [10,19,23,25,30,31], however, administration of 25D increases Sclerostin levels both in healthy subjects [32] and in CKD-5D [33] while in vitro experiments show that intracellular 1,25D stimulates Sclerostin production by osteocytes [34]. Therefore, in KTR, higher circulating 25D by allowing higher intracellular 1,25Dsynthesis might, in turn, favor Sclerostin synthesis by osteocytes. Importantly, the eventual increment of serum Sclerostin levels will exert a negative feed-back on 1,25D synthesis, in line with current physiological concepts. Thus, the inconsistency of our data with other clinical observations should be related to differences in ethnicity or therapies, including vitamin D supplementation that is mostly not specified in the available papers [10,11,18,19] while were not prescribed or timely withhold in our patients.

To our knowledge, also the positive relationship between Sclerostin and sKlotho observed in multivariate analysis in our KTR is new. We know that sKlotho interacts with Wnt pathway by either inhibiting Wnt ligands [35] or by upregulating Wnt inhibitors [36], thus allowing to accept the present correlation, which reinforces the potential clinical significance of sKlotho in renal patients as a marker of the dialogue between bone and the kidney.

**Fig 3:**
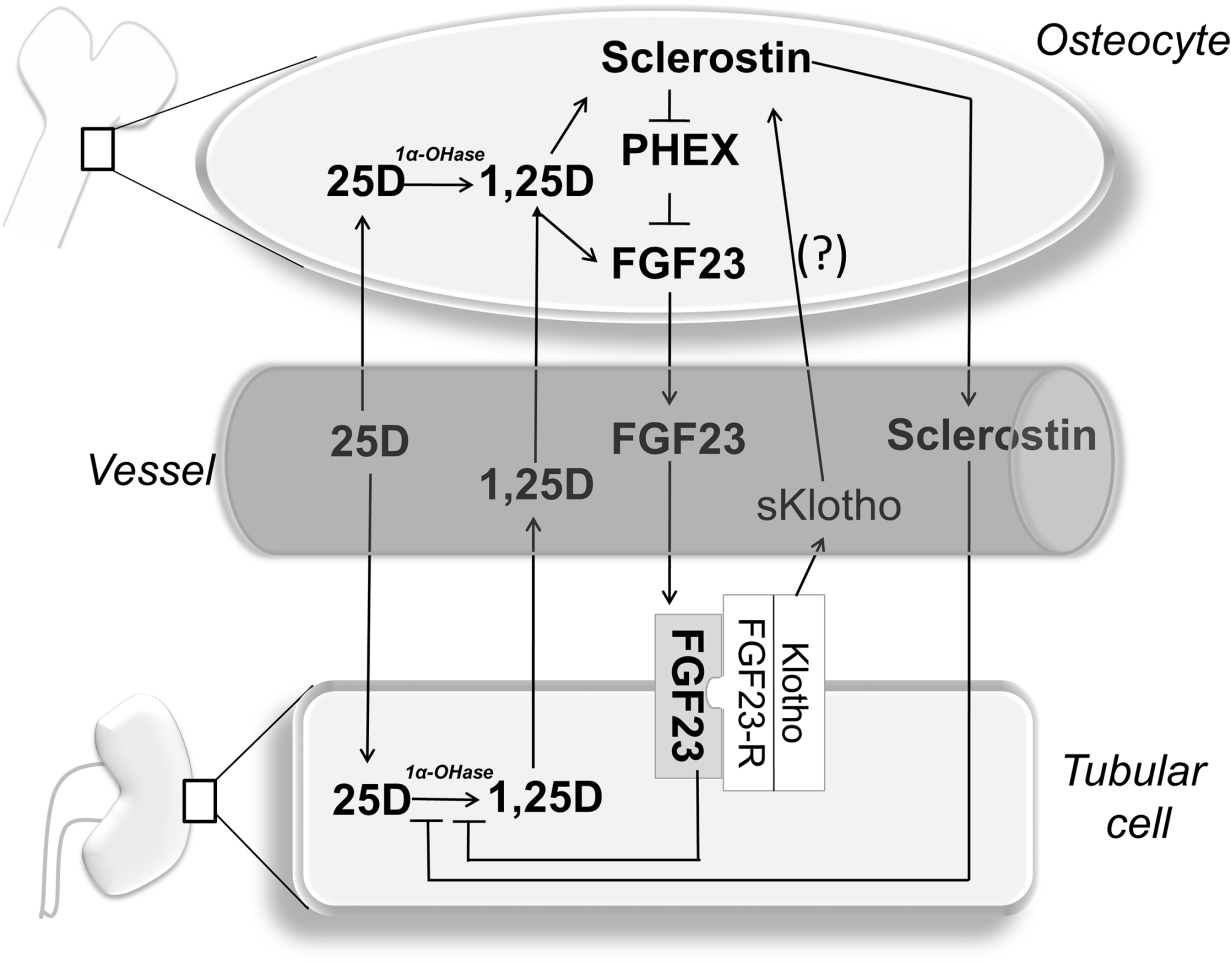
Bone-kidney axis. Schematic of the possible pathophysiologic links of Sclerostin with FGF23-Klotho and vitamin D system in kidney transplant patients. Intracellular 1,25D stimulates Sclerostin synthesis. Sclerostin, through inhibition of PHEX (an FGF23 inhibitor), indirectly increases FGF23 production. Also, Sclerostin inhibits 1,25D synthesis through direct (inhibition of renal 1-αOH-ase) and indirect (stimulation of FGF23) effects. S-Klotho could be another positive local modulator of Sclerostin synthesis. Abbreviations: PHEX, a protein encoded by the phosphate regulating gene with homologies to endopeptidases on the X chromosome; FGF23, Fibroblast growth factor 23; 1αOH-ase, 1alpha-hydroxylase; 25D, 25-hydroxyvitaminD; 1,25D, 1,25-dihydroxyvitaminD; Arrows: Stimulation; End Lines: Inhibition.

The complex interactions of Sclerostin with other divalent-ion regulating systems emerging from our results are tentatively illustrated in Fig 3. They deserve appreciation since they help describe, at least in part, the complex endocrine link existing between bone and the kidney.

As a whole, our data suggest that Sclerostin is involved not only with bone turnover, but also with the synthesis of other divalent ion regulating-hormones. Indeed, clinical observations reporting on Sclerostin correlations in different types of renal patients are incomplete and possibly inconsistent, as schematically recapitulated in Table 4. This table shows, first of all, that none of the available study contemporarily examines all the biomarkers we assayed. Second, that when present, the correlations with AP and PTH are consistently negative both in healthy and kidney patients. Third, that similarly consistent and of positive sign (when assayed and if present), are the correlations with serum phosphate and FGF23 but in renal patients only. Fourth, that the relationship with 25D (as a marker of vitamin D repletion) may vary in different clinical conditions, while the negative association with the circulating active hormone seems consistent in KTR. Importantly, this last association highlights the negative feed-back of Sclerostin, whose bone synthesis is induced by vitamin D, on 1,25D synthesis.

**Table 4:** Currently reported correlations (either linear or multivariate analysis) of serum Sclerostin levels in different clinical condition.

		Correlations										
Population	Sample size	eGFR	Ca	P	PTH	AP/ bALP	25D	1,25D	FGF23	sKlotho	CTX	Ref.
**Healthy subjects/ General population**	161		Neg*	*n*.*s*.	*n*.*s*.	*n*.*s*.	*n*.*s*.				*n*.*s*.	23
	40		*n*.*s*.	*n*.*s*.	Neg*^	*n*.*s*.	*n*.*s*.	*n*.*s*.			*n*.*s*.	25
	593			*n*.*s*.	*n*.*s*.	Neg*					Neg*	21
**ConservativeCKD**	90	Neg*^	*n*.*s*.	Pos*^	*n*.*s*.	*n*.*s*.	*n*.*s*.	.				10
	173	Neg*	*n*.*s*.	Pos*^	Pos*	*n*.*s*.	*n*.*s*.		**Pos^**			11
	140^§^		*n*.*s*.	Pos*^	*n*.*s*.		*n*.*s*.	*n*.*s*.	Pos*			16
**CKD-5D**	673	Neg*	Pos*	Pos*	Neg*	Neg*						14
	181		*n*.*s*.	*n*.*s*.	*n*.*s*.	Neg*						15
	60		Pos*	*n*.*s*.	Neg*							13
**KTR**	268	Neg*^	*n*.*s*.	*n*.*s*.	**Neg^**		*n*.*s*.	Neg*^				18
	31	**Neg^**	*n*.*s*.	**Pos^**	*n*.*s*.	*n*.*s*.	*n*.*s*.				*n*.*s*.	19
	80	*n*.*s*.	*n*.*s*.	*n*.*s*.	*n*.*s*.	Neg*^	Pos*^	**Neg^**	Pos*	**Pos^**	*n*.*s*.	Our KTR

**Abbreviations**: eGFR, estimated glomerular filtration rate; Ca, calcium; P, phosphate; PTH, parathyroid hormone; AP, alkaline phosphatase; bALP, bone alkaline phosphatase; 25D, 25-hydroxyvitaminD; 1,25D, 1,25-dihydroxyvitaminD; FGF23, Fibroblast Growth Factor 23; CKD-5D, dialysis patients; CTX, collagen type 1 cross-linked C-telopeptide; KTR, kidney transplant recipients. Empty boxes indicate absence of data.

(*) Univariate analysis

(^) Multivariate analysis

(^§^) 46 patients with CKD-5D.

In any case, our results highlights the complex role for Sclerostin in CKD-MBD in KTR and indicate a significant contribution to mineral homeostasis. From a systemic point of view, this complex involvement suggests potential links even with extra-skeletal calcification processes. In fact, Sclerostin is currently regarded as a promising new player in CKD-MBD, possibly implicated with the pathomechanisms of vascular calcification and cardiovascular mortality [14,18,20].

Several limitations can be recognized in our study. First, an increased number of cases would have reinforced the reliability of our results, however, our sample size is definitely adequate to obtain convincing preliminary correlations. Second, a more precise assessment of renal function (e.g. by inulin or iohexol clearance) would have strengthen our finding of no correlation with eGFR. For the same reason if we measured urinary Sclerostin we could have confirmed its increased secretion along with eGFR reduction. Third, reliability of commercial sKlotho assays has been recently questioned [37]. However, in the present study we employed the most accredited commercial method which, in our hands, evidenced early reduction since CKD stage 2 [38], in line with other published papers [39].

Strengths of our study are the contemporary assessment of a number of biomarkers and the biological plausibility of the relationships described.

## Conclusions

Serum Sclerostin levels in KTR are mostly normal and apparently influenced more by bone turnover than by GFR reduction. Sclerostin can be regarded as a novel negative biomarker of bone turnover, with complex interactions with other modulators of bone cells activity (FGF23/Klotho and Vitamin D in particular). As such, its role as a new player in the field of bone-kidney axis in CKD in general and in KTR in particular, is confirmed and seems to deserve further appreciation.
